# Analysis of social media use by European plastic surgery societies: A missing link for #PlasticSurgery

**DOI:** 10.1371/journal.pone.0258120

**Published:** 2021-10-14

**Authors:** Sebastian P. Nischwitz, Hanna Luze, Katharina Rauch, Benjamin T. Lemelman, Albrecht Heine-Geldern, Thomas Rappl, Alessandro Gualdi, Lars-Peter Kamolz, Andres A. Maldonado

**Affiliations:** 1 Division of Plastic, Aesthetic and Reconstructive Surgery, Department of Surgery, Medical University Graz, Graz, Austria; 2 COREMED – Cooperative Centre for Regenerative Medicine, JOANNEUM RESEARCH Forschungsgesellschaft mbH, Graz, Austria; 3 KAGES – The Healthcare Company of Styria, Graz, Austria; 4 Section of Plastic Surgery, Department of Surgery, Advocate Illinois Masonic Medical Center, Chicago, Illinois, United States of America; 5 Department of Plastic, Hand and Reconstructive Surgery, BG Trauma Center Frankfurt am Main, Academic Hospital of the Goethe University Frankfurt am Main, Frankfurt am Main, Germany; 6 Dental School, Vita-Salute San Raffaele University, Milan, Italy; 7 Department of Plastic Surgery, University Hospital Getafe, Madrid, Spain; Universita degli Studi della Campania Luigi Vanvitelli, ITALY

## Abstract

**Purpose:**

The field of Plastic Surgery is prominent on social media around the world. Board certified plastic surgeons and societies of plastic surgery play a role in providing accurate, evidence-based information to the public, patients, and colleagues. The aim of this study was to explore the use of social media by European Plastic Surgery Societies.

**Methods and materials:**

A retrospective analysis of the presence and activity of European Plastic Surgery Societies on Facebook, Twitter and Instagram was conducted between December 12^th^ 2018 and December 12^th^ 2019. The results have been compared to the American Society of Plastic Surgeons.

**Results:**

Twenty, eleven and nine European societies yielded an active account on Facebook, Twitter and Instagram respectively. Only seven European societies had an account on all three platforms and were therefore considered *polypresent*. The amount of followers of those seven societies was significantly higher than of the others (p-value = 0.02). Their activity yielded significantly more posts on Facebook (p-value = 0.02). The American Society of Plastic Surgeons had more followers on all three platforms than all European societies combined.

**Conclusion:**

Social media are still rather unexploited by European Plastic Surgery Societies. A tendency towards increased visibility can be observed, yet a higher penetration is required to further educate and engage through social media. The quantitative data provided serve as reasonable foundation for further studies and a guide for growth of #PlasticSurgery.

## Introduction

Plastic surgeons relied on referrals, reputation and academic pedigree to attract new patients for the past decades [1, 2]. The influence of adverts or television programs on people’s consideration of plastic surgery has steadily increased [3]. With the advancement of technology, social media has become an important means of communication [4]. The number of worldwide active social media users is constantly growing and is expected to reach 3.02 billion users by 2021, around one-third of Earth’s population [5]. Also within plastic surgery, social media has become more and more important and used. Patients considering procedures increasingly rely on easily accessible information provided by social media and search engines [6, 7], hence the popularity among plastic surgeons has also increased. Plastic Surgery has become the most present medical specialty on social media [4, 8], seizing its distinction as pioneer among medical specialties [9]. The platforms are used as marketing tool to attract new patients but also as reliable communication platform to efficiently exchange information between professionals [10]. However, the functions provided by digital tools are tempting [11], and these platforms must be used thoughtfully and with all professional, legal, and ethical considerations to prevent trivialisation of medical procedures [12]. Although certain medical societies have released guidelines on the use of social media, plastic surgery, with its inherent visual nature and potential for sensationalism, could benefit from increasing direction regarding ethical use thereof [13]. Surgeons within the community are calling for more structured oversight and guidance regarding social media [14]; while Cho *et al*. formulate recommendations on the use of social media for young plastic surgeons [15], Economides *et al*. express the need for specific social media training in certain curricula [16]. Several studies have shown the popular use of the hashtag #PlasticSurgery within social media among public, celebrities or cosmetic practitioners [17, 18]. Considerably few posts are connected to academic institutions, scientific journals, or board-certified surgeons [18, 19]. The widespread use of the hashtag and the tendency to consult the internet for information about plastic surgeries, calls for thorough filtering to guarantee safety for patients and education for colleagues. Social media misuse by medical professionals is unfortunately no exemption, and has been sanctioned by medical boards [20, 21]. Besides the prevention of misuse, we want to emphasize the importance of promotion of accurate information and science in the sense of the psychological concept of reinforcement. Plastic surgery societies (PSS) as stakeholders of their respective members are well-eligible entities to fulfil this function, support scientific appropriate information and provide education and leadership for their members. By proper and purposeful use of social media, additional responsibility can be taken to clear misconceptions, improving patients’ education and offering support in decision-making to improve safety. The American Society of Plastic Surgeons (ASPS) has recognized social media as powerful tool to engage and educate [17, 22]; to our knowledge no investigation has been conducted about the use of social media by PSS in Europe.

The aim of this study was to analyse European PSS’ use of three of the most popular social media (Facebook, Twitter, Instagram), and to compare it to ASPS.

## Methods and materials

A retrospective analysis of the social media activity of European PSS within one year (December 12^th^ 2018 to 2019) was performed. European countries have been screened for their existence of a PSS. The social media platforms Facebook, Twitter and Instagram have been searched for an account of the respective society and the activity thereof. ASPS has been used as comparison. The data collection method complied with the terms and conditions for the respective website.

### Selection of PSS

All countries have been extracted from the online website of the World Health Organization (WHO). While this list reflects not necessarily countries located in Europe, it comprises all countries in the jurisdiction of the WHO Regional Office for Europe. For better understanding, “European countries” or “Europe” further includes all countries in that list. A Google Search using the term “Plastic Surgery Society” + [the respective country name in English] has been performed. If the results yielded an unambiguous society/association, it was included. If there was doubt, or several societies showed up, the one listed first and clearly stated “Plastic Surgery” was chosen. The list was compared the national societies presented by the European Association of Plastic Surgeons (EURAPS).

### Search algorithm on social media

Facebook, Twitter and Instagram were searched for an account of the respective society. For every search query the society’s full name, official abbreviation, and (if applicable) name in the respective mother tongue was used. The number of followers and the number of active posts/tweets in the study period (excluding simple picture changes or re-posts/re-tweets) were analyzed. Five-digit numbers were rounded down to the hundred.

### Data analysis

PSS have been categorized as *polypresent* when present on all three platforms, and as *present* when present on one or two platforms. The data were summarized using absolutes and percentages of the total sum for categorical variables. Arithmetic means were used for continuous variables. Unpaired t-test was used to determine statistical significance with the level of significance being set to p-value <0.05.

## Results

### Plastic surgery societies

**Fig 1:**
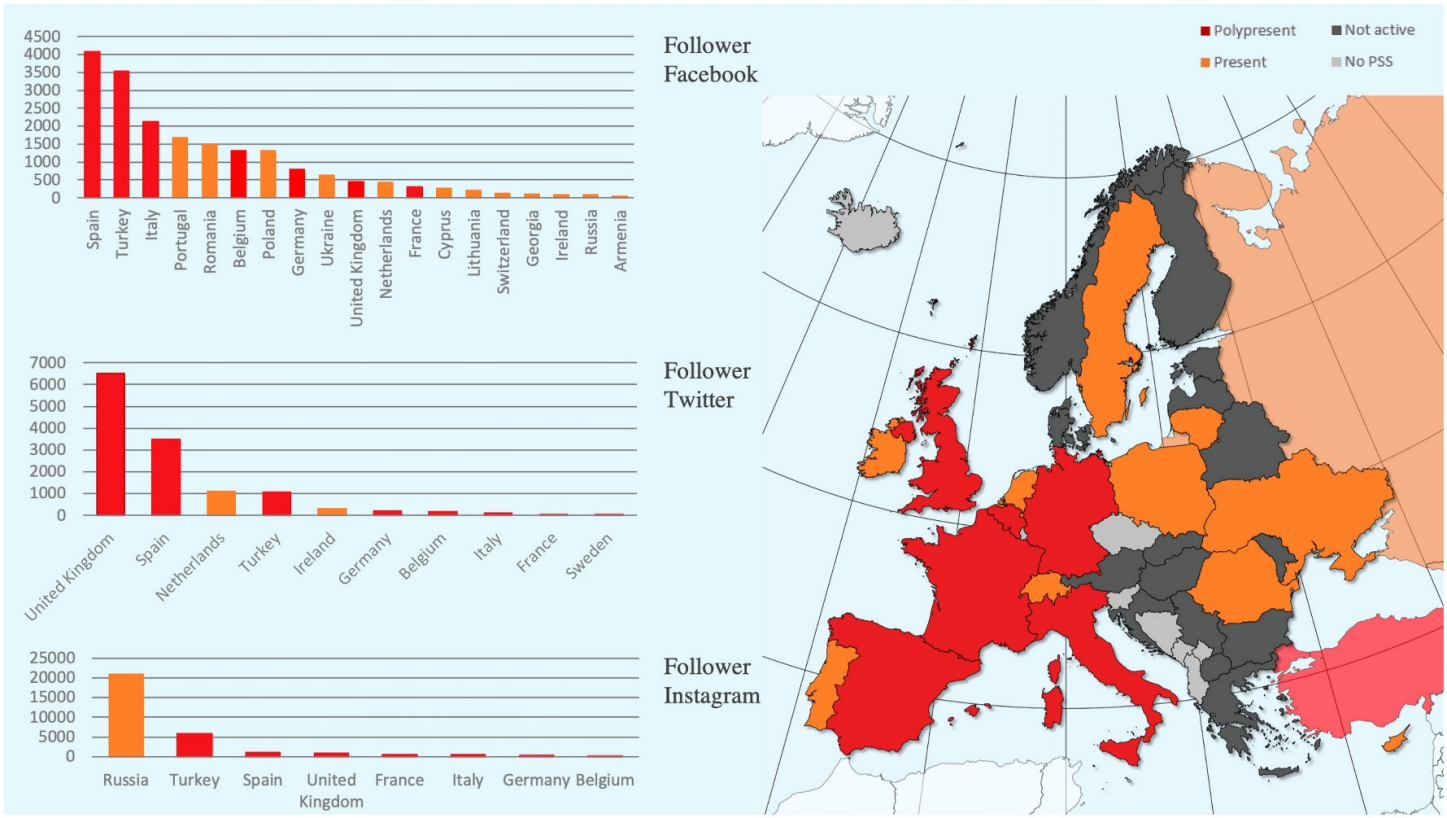
Visual representation of the results. European countries without PSS (light grey), with a PSS but no presence on social media (dark grey), a present PSS (account on one or two platforms; ochre), a polypresent PSS (account on all three platforms; red). Pale-coloured countries are located outside of Europe geographically. The graphic was created using Magic Maps 2 for Mac (Version 2.3.6; © 2010–2020 Evan Miller).

Fifty-four European countries, the USA and Europe have been screened for the availability of a PSS. We found a PSS for 41 (73.21%) countries (39 national, EURAPS and ASPS, see Fig 1 and Table 1).

**Table 1:** Plastic surgery societies.

Country name	PSS [English]	Acronym	Active on		
			Facebook	Twitter	Instagram
Albania	n/f	-	-	-	-
Andorra	n/f	-	-	-	-
Armenia	Armenian Association of Plastic, Reconstructive and Aesthetic Surgeons	AAPRAS	Y	N	N
Austria	Austrian Society for Plastic, Aesthetic and Reconstructive Surgery	OEGPAERC	N	N	N
Azerbaijan	Society of Plastic Surgery Azerbaijan	PCİB	N	N	N
Belarus	Belarusian Society of Plastic Surgeons	-	N	N	N
Belgium	Royal Belgian Society for Plastic Surgery	RBSPS	Y	Y	Y
Bosnia & Herzegovina	n/f	-	-	-	-
Bulgaria	Bulgarian Association of Plastic, Reconstructive and Aesthetic Surgery	BULAPRAS	N	N	N
Croatia	Croatian Society of Plastic, Reconstructive and Aesthetic Surgery	HDPREK	N	N	N
Cyprus	Cyprus Society of Plastic Reconstructive & Aesthetic Surgery	CySPRAS	Y	N	N
Czechia	n/f	-	-	-	-
Denmark	Danish Society for Plastic and Reconstructive Surgery	DSPR	N	N	N
Estonia	Estonian Society for Plastic and Reconstructive Surgery	EPRS	N	N	N
Finland	Finnish Association of Plastic, Reconstructive and Aesthetic Surgeons	FAPRAS	N	N	N
France	French Society of Plastic, Reconstructive and Aesthetic Surgery	SoFCPRE	Y	Y	Y
Georgia	Georgia Society of Plastic Surgeons	GSPS	Y	N	N
Germany	German Society of Plastic, Reconstructive and Aesthetic Surgeons	DGPRAEC	Y	Y	Y
Greece	Hellenic Society of Plastic Reconstructive and Aesthetic Surgery	HESPRAS	N	N	N
Hungary	Hungarian Society of Plastic, Reconstructive and Aesthetic Surgery	HSPRAS	N	N	N
Iceland	n/f	-	-	-	-
Ireland	Irish Association of Plastic Surgeons	IAPS	Y	Y	N
Israel	Israeli Society of Plastic & Aesthetic Surgery	-	N	N	N
Italy	Italian Society of Plastic, Reconstructive & Aesthetic Surgery	SICPRE	Y	Y	Y
Kazakhstan	Kazakhstan Society of Aesthetic and Plastic Surgery	NSAPS	N	N	N
Kyrgyzstan	n/f	-	-	-	-
Latvia	Latvian Association of Plastic Surgeons	-	N	N	N
Liechtenstein	n/f	-	-	-	-
Lithuania	Lithuanian Society of Plastic and Reconstructive Surgery	PRCH	Y	N	N
Luxembourg	n/f	-	-	-	-
Macedonia	Macedonian Association of Plastic, Reconstructive and Aesthetic Surgeons	MAPRAS	N	N	N
Malta	n/f	-	-	-	-
Monaco	n/f	-	-	-	-
Montenegro	n/f	-	-	-	-
Netherlands	Netherlands Society for Plastic Surgery	NVPC	Y	Y	N
Norway	Norwegian Plastic Surgery Association	-	N	N	N
Poland	Polish Society of Plastic, Reconstructive and Aesthetic Surgery	PTCHPRIE	Y	N	N
Portugal	Portuguese Society of Reconstructive and Aesthetic Plastic Surgery	SPCPRE	Y	N	N
Republic of Moldova	Moldavian Association of Plastic, Reconstructive and Aesthetic Surgery	-	N	N	N
Romania	The Association of Plastic Surgeons from Romania	ACPR	Y	N	N
Russia	Russian Society of Plastic, Reconstructive and Aesthetic Surgery	SPRAS	Y	N	Y
San Marino	n/f	-	-	-	-
Serbia	Serbian Association for Plastic Reconstructive and Aesthetic Surgery	SRBPRAS	N	N	N
Slovakia	Slovak Society Plastic and Aesthetic Surgery	SSPAS	N	N	N
Slovenia	n/f	-	-	-	-
Spain	Spanish Society of Plastic, Reconstructive and Aesthetic Surgery	SECPRE	Y	Y	Y
Sweden	Swedish Plastic Surgery Association	SPKF	N	Y	N
Switzerland	Swiss Plastic Surgery Association	SGPRAC	Y	N	N
Tajikistan	n/f	-	-	-	-
Turkey	Turkish Plastic Reconstructive and Aesthetic Surgery Association	TRPECD	Y	Y	Y
Turkmenistan	n/f	-	-	-	-
Ukraine	Ukrainian Society of Aesthetic Plastic Surgeons	USAPS	Y	N	N
United Kingdom	British Association of Plastic, Reconstructive and Aesthetic Surgeons	BAPRAS	Y	Y	Y
Uzbekistan	Society of Plastic, Reconstructive and Aesthetic Surgeons of Uzbekistan	-	N	N	N
Europe	European Association of Plastic Surgeons	EURAPS	Y	Y	Y
USA	American Society of Plastic Surgeons	ASPS	Y	Y	Y
**56 countries**	**41 PSS**		**21 Y**	**12 Y**	**10 Y**

Representative Plastic Surgery Societies (PSS) of all European countries, USA, and their respective activity on social media. Y = yes, N = no, n/f = none found.

### Social media portfolio

Twenty out of 39 (51.28%) national PSS had an active account on at least one social media platform. Except Sweden, all of these had a Facebook account. Seven out of the 20 national PSS (35.00%; 17.95% of European PSS) yielded a social media account on all three platforms, and were therefore categorized as *polypresent* (Belgium, France, Germany, Italy, Spain, Turkey, United Kingdom). Three national PSS (15.00%) showed a social media presence on two, and ten (50.00%) on one platform. Those 13 national PSS were categorized as *present*.

**Fig 2:**
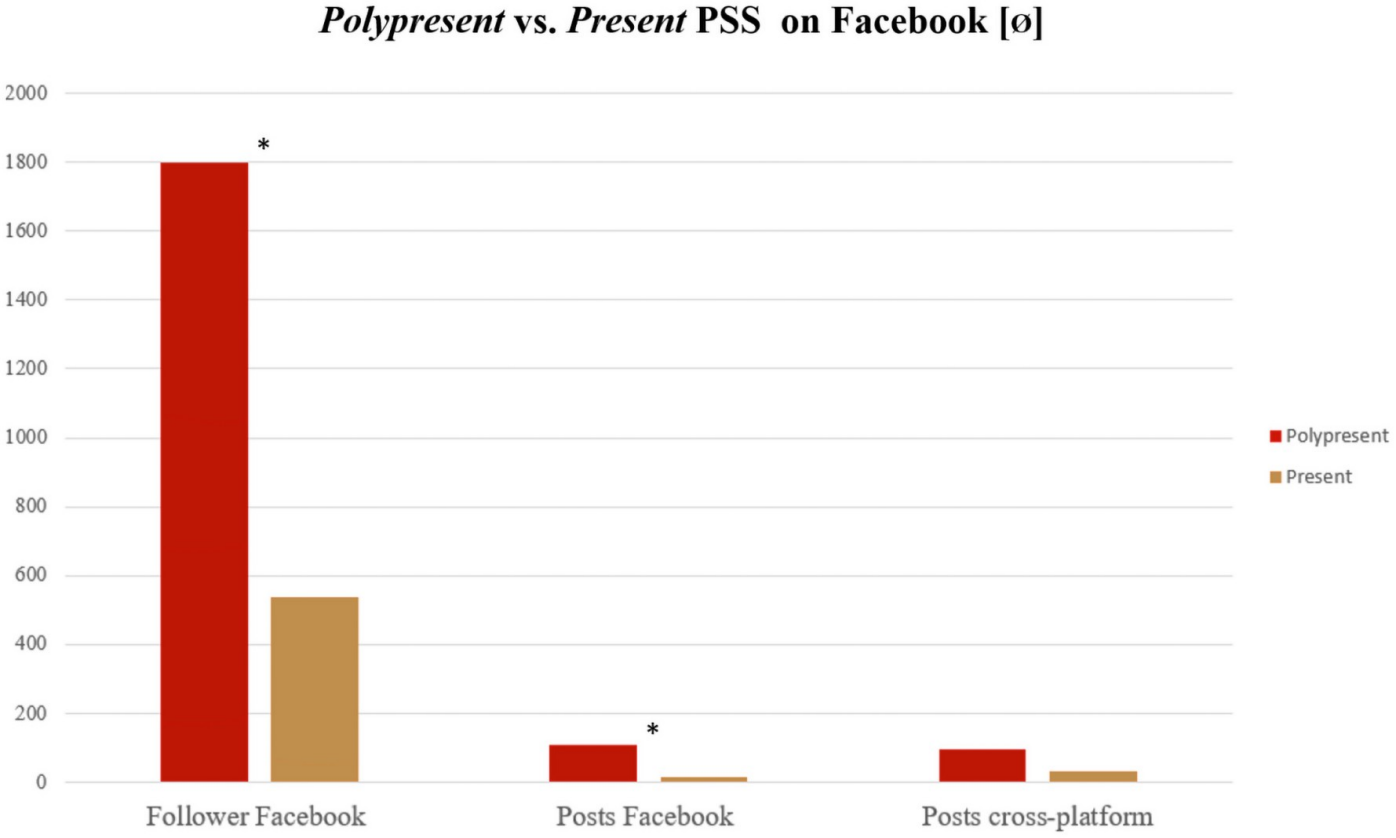
Status of PSS. Comparison between polypresent (account on all three platforms; red) and present (account on one or two platforms; ochre) PSS. A significant difference can be seen in Facebook followers and posts. The cross-platform activity (posts) shows no significant difference.

The amount of Facebook followers significantly differed between *polypresent* and *present* PSS (p-value = 0.0184). The amount of Facebook posts also significantly differed between *polypresent* and *present* PSS (p-value = 0.0225). No statistically significant difference was seen between *polypresent* and *present* PSS when looking at the amount of all posts cross-platform (p-value = 0.06). Fig 2 shows a comparison between *polypresent* and *present* PSS social media portfolio.

### Facebook activity

Facebook activity is summarized in Table 2.

**Table 2:** Active PSS.

Country name	PSS [Acronym]	Facebook		Twitter		Instagram	
		Follower (%)	Posts (%)	Follower (%)	Posts (%)	Follower (%)	Posts (%)
Armenia	AAPRAS	48 (0.23)	0	n/a		n/a	
Belgium	RBSPS	1314 (6.30)	24 (2.52)	158 (1.22)	1 (0.15)	127 (0.42)	7 (0.84)
Cyprus	CySPRAS	269 (1.29)*	3 (0.31)*	n/a		n/a	
France	SoFCPRE	317 (1.52)	6 (0.63)	27 (0.21)	0	536 (1.76)	5 (0.60)
Georgia	GSPS	107 (0.51)	1 (0.10)	n/a		n/a	
Germany	DGPRAEC	793 (3.80)	10 (1.06)	193 (1.48)	5 (0.74)	369 (1.21)	0
Ireland	IAPS	90 (0.43)	3 (0.31)	310 (2.38)	0	n/a	
Italy	SICPRE	2122 (10.18)	166 (17.40)	95 (0.73)	0	430 (1.41)*	39 (4.68)*
Lithuania	PRCH	216 (1.04)	17 (1.78)	n/a		n/a	
Netherlands	NVPC	421 (2.02)	8 (0.84)	1096 (8.43)	35 (5.18)	n/a	
Poland	PTCHPRIE	1313 (6.30)	17 (1.78)	n/a		n/a	
Portugal	SPCPRE	1675 (8.03)	16 (1.68)	n/a		n/a	
Romania	ACPR	1491 (7.15)	0	n/a		n/a	
Russia	SPRAS	82 (0.39)	40 (4.61)	n/a		**20900 (68.71)**	210 (25.18)
Spain	SECPRE	**4078 (19.56)**	**315 (33.02)**	3489 (26.83)	106 (15.68)	1079 (3.55)*	87 (10.43)*
Sweden	SPKF	n/a		26 (0.20)	42 (6.21)	n/a	
Switzerland	SGPRAC	116 (0.56)	5 (0.52)	n/a		n/a	
Turkey	TRPECD	3531 (16.93)	214 (22.43)	1076 (8.27)	**249 (36.83)**	5872 (19.31)	**393 (47.12)**
Ukraine	USAPS	624 (2.99)	73 (7.65)	n/a		n/a	
United Kingdom	BAPRAS	439 (2.11)	10 (1.05)	**6523 (50.16)**	238 (35.21)	855 (2.81)*	84 (10.07)*
Europe	EURAPS	1807 (8.66)	22 (2.31)	11 (0.09)	0	249 (0.82)	9 (1.08)
**Subtotal**		**20853 (100)**	**954 (100)**	**13004 (100)**	**676 (100)**	**30417 (100)**	**834 (100)**
USA	ASPS	86298 (413.84)	457 (47.90)	29700 (228.39)	1036 (153.25)	32900 (108.16)	607 (72.78)
**Total**		**107151 (513.84)**	**1411 (100)**	**42704 (328.39)**	**1712 (253.25)**	**63317 (208.16)**	**1441 (172.78)**

Representative Plastic Surgery Societies (PSS) active on Social Media. n/a = not active,

* = joined platform during study period, RAI = Relative Activity Index, Maxima printed in **bold**.

Twenty of the 40 representative European PSS yielded a Facebook account; in total 20853 people followed all 20 PSS. The average follower number of the PSS accounts was 1042.65 (range: 48 (Armenia, 0.23%)– 4078 (Spain, 19.56%)). Fig 2 shows the shares of European PSS in terms of Facebook followers.

There have been 954 posts by the 20 PSS. On average, they produced 47.70 posts (0.13 per day; range: 0 (Armenia, Romania)– 315 (Spain, 33.02%, 0.86 posts per day)).

20 of all 21 (Europe + ASPS) societies (95.2%) created their account before the study period. Only the Cyprus Society of Plastic Reconstructive & Aesthetic Surgery joined Facebook in June 2019.

**Fig 3:**
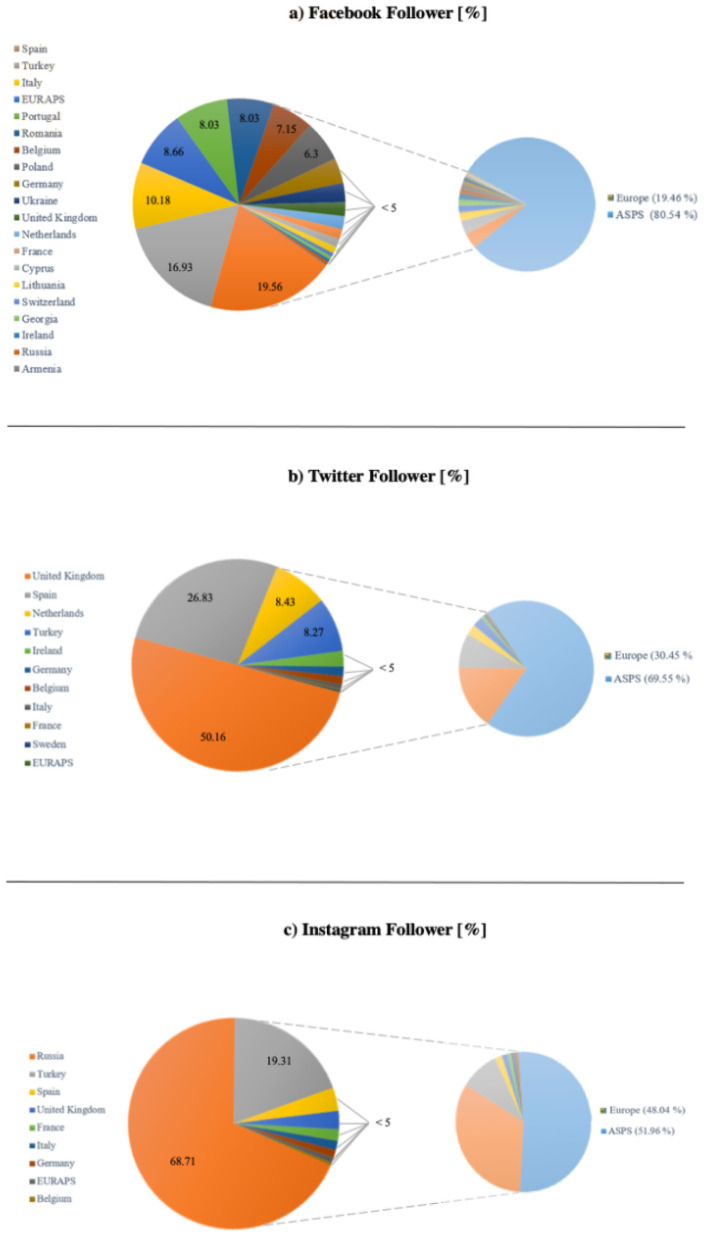
Social media followers of PSS. Share of (a) Facebook, (b) Twitter, and (c) Instagram followers of European PSS (left). On the right side, all of Europe is set in contrast to ASPS. Values are indicated in %.

**Fig 4:**
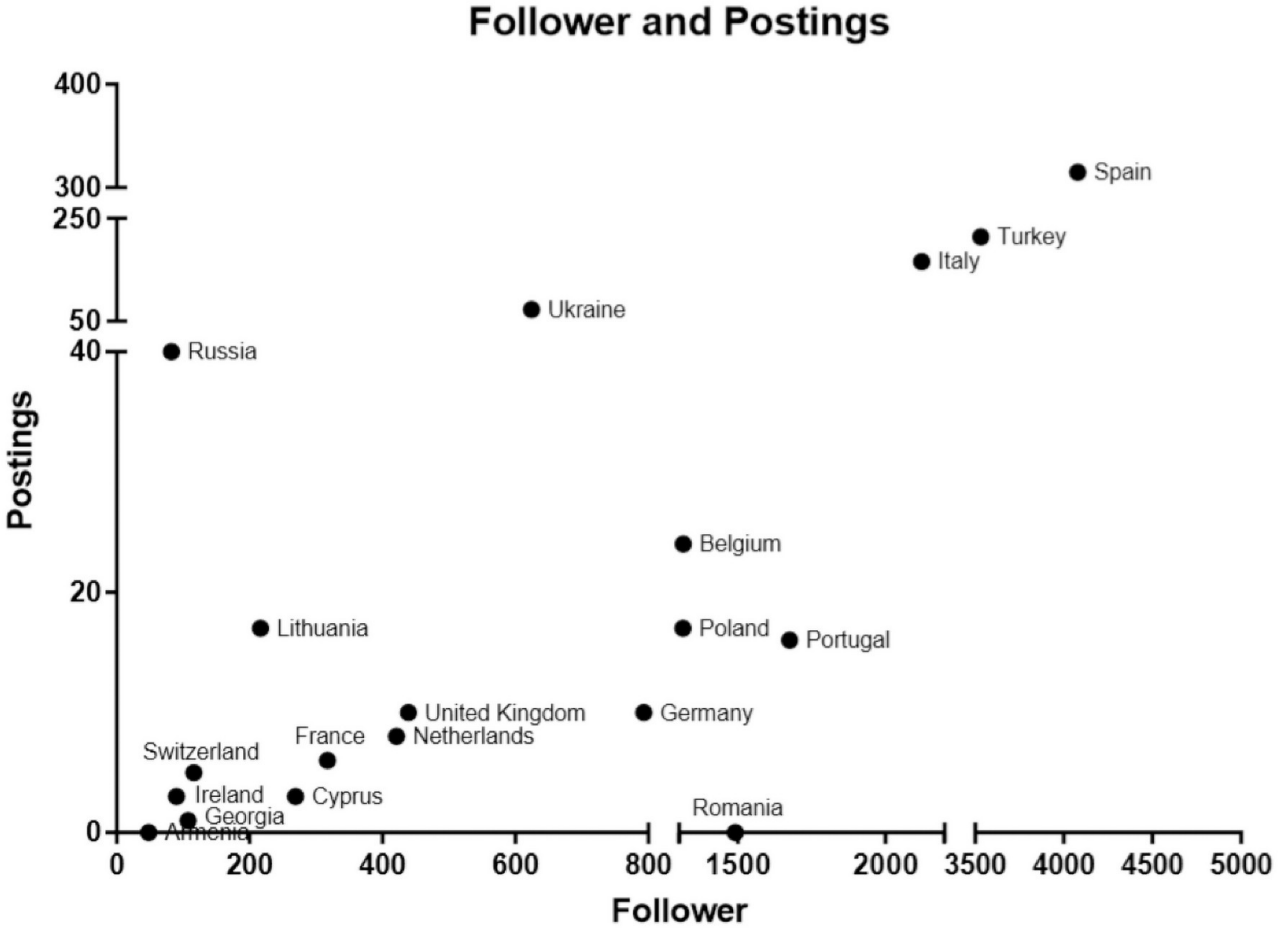
Comparison of European PSS Facebook activity. Followers (x-axis) vs. posts (y-axis) show a mostly linear distribution. Polypresent PSS are highlighted with an asterisk (*).

Fig 3a shows the shares of the respective European PSS in terms of Facebook followers. Fig 4 shows a graph of the European PSS’ Facebook activity with most of the PSS yielding a linear distribution of followers vs. posts.

### Twitter activity

Twitter activity is summarized in Table 2.

Eleven of the 40 PSS (27.5%) yielded a Twitter account, having 13004 followers (100%). The average number of followers was 1182.18 (range: 11 (EURAPS, 0.09%) - 6523 (UK, 50.16%)). Fig 3b shows the shares of the respective European PSS in terms of Twitter followers.

The 11 PSS posted 676 tweets (100%), averaging at 61.45 tweets per PSS (0.17 per day; range: 0 (Europe, France, Ireland, Italy)– 249 (Turkey, 36.83%, 0.68 tweets per day)).

All PSS joined Twitter before December 12^th^ 2018.

### Instagram activity

Instagram activity is summarized in Table 2.

Nine of the 40 PSS yielded an Instagram account (22.5%) having 30417 followers (100%). The average number of followers was 3379.67 (range: 127 (Belgium, 0.20%) - 20900 (Russia, 68.71%)). Fig 3c shows the shares of the respective European PSS in terms of Instagram followers.

The 9 PSS produced 834 posts (100%), averaging at 92.67 per account (0.25 posts per day; range: 0 (Germany)– 393 (Turkey, 47.12%, 1.08 posts per day)).

6 of the 9 PSS joined Instagram before the study period (77.78%). Spain (SECPRE, 01/2019), UK (BAPRAS, 02/2019) and Italy (SICPRE, 09/2019) joined Instagram during the study period and contributed 1079 (3.55%), 855 (2.81%) and 430 (1.41%) followers, and 87 (10.34%), 84 (10.07%) and 39 (4.68%) active posts, respectively.

### Comparison ASPS vs. Europe

ASPS is the most active society on all three social media platforms. Compared to the European countries (+EURAPS as neglectable variable) the ASPS held 413.84% (Facebook), 228.39% (Twitter) and 108.16% (Instagram) of followers. The ASPS was responsible for 457 (47.90%, Facebook), 1036 (153.25%, Twitter) and 607 (72.78%, Instagram) of posts compared to Europe (+EURAPS), totalling to 91.31% posts cross-platform.

**Fig 5:**
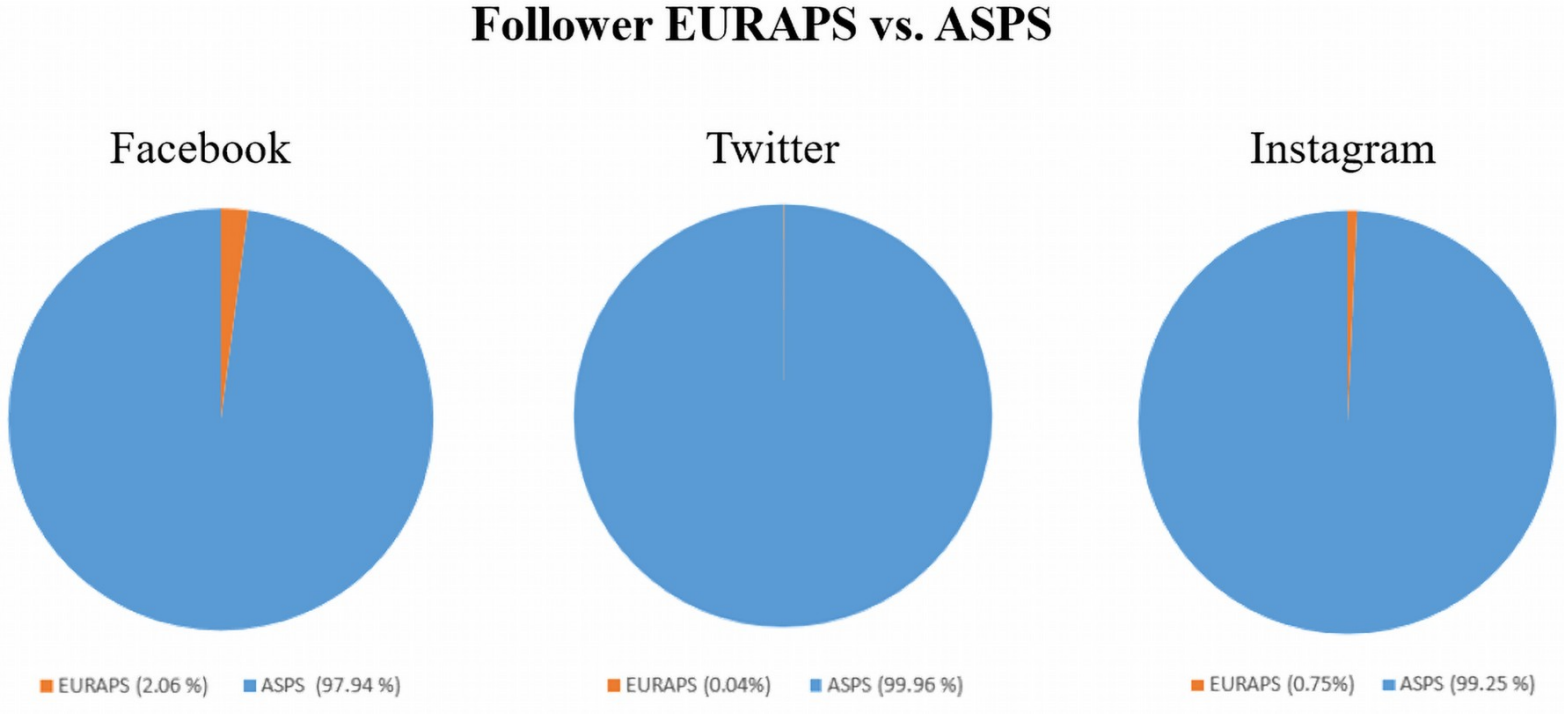
EURAPS vs. ASPS. Comparison between EURAPS and ASPS share of followers on all three social media platforms.

When being compared to EURAPS, as European umbrella organisation, the ASPS had 72 times the number of followers (7203.58%) and 67 times the number of posts (6774.19%) cross-platform. Fig 5 shows the comparison of EURAPS and ASPS in terms of followers. ASPS holds 97.94%, 99.96% and 99.25% of followers on Facebook, Twitter and Instagram, whereas EURAPS accounts for 2.06%, 0.04% and 0.75% respectively.

## Discussion

PSS serve as stakeholder of their respective members. That comprises a proper and respectable appearance and an adequate representation to the general public. In times of internet and social media, the latter have become a favoured means of communicating, expressing one’s feelings and/or beliefs, or just as simple amusement. Social media’s growing significance is a valuable tool for PSS to broadcast and promote news, insights and trending information as well as providing scientific accurate information, education and engagement [8].

We investigated the activity of European national PSS and EURAPS on Facebook, Twitter, and Instagram, assessing their presence, number of followers, and active posts in a 1-year period. The results were compared to the ones of the ASPS. To the authors’ information, this exploration was conducted for the first time.

We found at least one PSS for almost 75% of the countries. This represents a passable ratio, given the fact, that several European countries are rather small countries regarding inhabitants (e.g. Andorra with 77000 inhabitants [23]) or are ranked distinctly below the world’s average gross domestic product per capita (e.g. Albania 5373US$ [24]), possibly indicating a low need for a PSS.

We have found Facebook to be the most used social medium (20 accounts), followed by Twitter (11 accounts), and Instagram (9 accounts). Seven national PSS (+EURAPS) were present on all three platforms (Belgium, France, Germany, Italy, Spain, Turkey, UK, EURAPS). The newest social medium (Instagram, founded 2010) shows to be the most used platform with 3379.67 followers per account, followed by Twitter (1182.18 followers per account) and Facebook (1042.65 followers per account). More interestingly, 30% of the active Instagram accounts have not even existed, at the beginning of the investigation’s inclusion. This displays the current trend in social media, wherein Instagram has generally gained the most popularity and users recently (2016: ~500m, 2018: ~1bn [25]). Facebook, as oldest of the three platforms (founded 2004) still accounts the highest absolute user number (2.5bn users [5]); representing our findings in terms of active accounts, but not in terms of followers (20853 vs. 30417 on Instagram), and comes even last regarding the followers per account. Several studies [7, 26] were able to show Facebook to be *the* go-to platform with most users and engagement for plastic surgical content, calling Europe to action to increase their coverage and providing the needed assistance. *Polypresent* PSS had significantly more followers than *present* PSS. However, the variable that is influenceable by the PSS, the active posts, did not significantly differ cross-platform. This suggests that having a far-reaching social media portfolio across platforms may increase a society’s reach within Facebook, as seen in the increased number of followers.

When looking at the number of active posts, Facebook yielded the most (954), followed by Instagram (834), and Twitter (676). Interestingly, when examining the ASPS, Twitter is the most used platform with 1036 active tweets (vs. 607 Instagram–and 457 Facebook posts). This might be due to the characteristics and nature of tweets, only allowing a certain number of characters as well as its focus not being on a graphical, but rather informational content, possibly appealing to a more scientific-oriented audience, hence providing a better platform for inter-professional exchange [27, 28]. When comparing the number of posts per PSS per day between Facebook (0.13), Twitter (0.17), and Instagram (0.25), Facebook is again the platform with the lowest amount. Same data were found when looking at the ASPS (1.25, 2.84, and 1.66 posts per day respectively). A reason therefore, besides the before mentioned perks of Twitter, might be Facebook’s general popularity, tempting a PSS to create an account without actually being active on the platform. Instagram’s popularity might be caused by the rising significance of appearance and aesthetics, and Plastic Surgery appears to be aligned with it and its unique focus on visuals [11]. Particularly patients seeking aesthetic surgery, as a growing part of plastic surgery, tend to gather information on social media prior to consultations [26, 29]; gaining a first impression of the surgeon as well as browsing through pictures of surgical results can be done from the comfort of one’s own home. A recent study by Fan *et al*. even revealed Facebook to be the single most influential online method of selecting a plastic surgeon in people under the age of 35 [7]. Several recent studies have investigated the involvement of individual medical professionals in social media and could show a steady increase [30–32] With social media having become a valuable marketing tool, more and more plastic surgeons created professional profiles to attract patients and promote themselves; plastic surgeons tend to welcome these platforms in particular due to the inherent medial popularity of their specialty [33]. Yet, one needs to consider the ethical ambiguity arising from different marketing strategies and legal prerequisites in different countries by using pictures showing patients to promote oneself. Due to the growing community on social media, it is reasonable for the surgeons’ stakeholders, the PSS, to expand their horizon, join these platforms, and back their affiliated surgeons. A serious and diligent online presence in a widespread portfolio may help to promote serious and scientific plastic surgery, to prevent trivialization of especially aesthetic procedures [12]; and health professionals on social media could improve the quality of information and help to suppress “fake-news” [34]. Since marketing is not as important in general plastic surgery as in aesthetic surgery, understandably the analysed PSS are emerging slowly to social media as opposed to more aesthetic-oriented societies or surgeons [35]. Further studies should compare the social media presence and activity of different medical specialties and individual health professionals to evaluate possible correlations. This reflects the general acceptance of social media by patients and doctors to exchange and connect on social media, leaving an incredible opportunity for PSS to strengthen their position and community. However, one needs to cautiously evaluate, whether a presence on social media is merely to attract future customers or to reach online communities to spread information.

The United States, as birthplace of five of the six most popular social media platforms, has always acted as one of the pioneers when it comes to digitalisation and information technology [5] and Branford *et al*. concluded in 2016 already, that ASPS has recognized social media’s potential as valuable tool to educate and engage [17]. Given the fact, that it is the largest country’s PSS investigated (and the largest society), they understandably hold 41 times, almost 23 times and almost 11 times the amount of followers compared to Europe on Facebook, Twitter, and Instagram respectively. They account for 47.90%, 153.25% and 72.78% of posts compared to Europe, showing a rather large discrepancy between share of followers and share of posts, especially on Facebook. This leads to the assumption that a “successful” social media portfolio does not depend on the amount of active posts but on the number of followers [19]. We believe that the presence on platforms is of utmost importance to gain followers. Our data showed that the amount of followers on Facebook as *the* go-to platform [7, 26] is significantly different between *polypresent* and *present* PSS. The overall high numbers of the ASPS compared to EURAPS emphasize the value of a social media taskforce (or “digital case manager” as advocated in other specialties as well [34, 36]), which should be implemented in EURAPS to further increase the outreach.

While ASPS still holds a paramount position in social media activity, European nations’ PSS are also starting to exploit the potential of these web-based communication platforms. Their use and coverage are an effective and easy-to-use way to promote serious, and scientific plastic surgery, also increasing the safety for prospective patients. This almost renders it obligatory for PSS to further engage in social media. The recent pandemic further forced the medical community to the online world, so that PSS as stakeholders of plastic surgeons should appoint their taskforces, foster their presences, promote scientific information and advanced trainings to seize and profit from increased and homogeneous knowledge and (inter-)national exchange. With the data provided, further qualitative as well as impact-oriented studies can help to engage and consolidate the position of PSS as up-to-date stakeholders of plastic surgeons worldwide.

One of the study’s limitation is the sole exploratory nature of the conducted investigation. We screened quantity and not quality of posts, thus actual content was not evaluated; and activity does not equal quality. Yet, we deliver valuable results as basis for further studies to investigate quality and content of the plastic surgery stakeholders’ social media activity. This can lead to additional analyses to evaluate the economic impact of social media ‘marketing’ on plastic surgery or the progression of growth within respective countries. Another limitation is the possible selection bias. While we tried selecting the PSS most likely to represent general plastic surgery, possibly no adequate web presence of a more suitable PSS was found. Furthermore, a factor influencing ASPS’ numbers to be tenfolds higher compared to the European ones, is the European federalism. Lastly, in the world of online activity the possibility of manipulation and/or hoaxes can never be eliminated, leading to possible wrong numbers of followers or selection of unofficial accounts [19, 37].

## Conclusion

The use of social media is steadily increasing, emphasizing their significance as communication tools and sources of information. Plastic surgery, as in all fields of medicine, has many online myths promoted by charlatans. Plastic surgeons and the societies have an opportunity to promote evidence-based practice and scientific advancements through social media. The present study has demonstrated a low penetration of the market by European PSS compared to the ASPS in the USA. However, a trend towards increased visibility can be observed, especially in the larger societies of the UK, Spain and Italy. Our data show that a) coverage on social media, measured by the amount of Facebook followers as highest-penetrating social media platform is significantly higher in *polypresent* vs. *present* PSS; b) the cross-platform activity was not significantly differing. Therefore, we believe the presence on social media is a valuable first step for broadened outreach. ASPS showed higher numbers compared to EURAPS, suggesting the need for a social media task force to address education and patient safety in Europe. The quantitative data provided serve as reasonable foundation for further studies. Social media are still a nebulous tool for European PSS, whose potential remains to be reached.

## Supporting information

S1 Dataset (XLSX)
